# Inorganic and Organic Nitrogen Acquisition by a Fern *Dicranopteris dichotoma* in a Subtropical Forest in South China

**DOI:** 10.1371/journal.pone.0090075

**Published:** 2014-05-14

**Authors:** Xingliang Xu, Qingkang Li, Jingyuan Wang, Leiming Zhang, Shengni Tian, Lin Zhi, Qianru Li, Yue Sun

**Affiliations:** 1 Key Laboratory of Ecosystem Network Observation and Modeling, Institute of Geographic Sciences and Natural Resources Research, Chinese Academy of Sciences, Beijing, China; 2 Graduate School of the Chinese Academy of Sciences, Beijing, China; 3 College of Life Science, Anhui Agriculture University, Hefei, Anhui Province, China; 4 Xikou Junior Middle School, Xikou City, Zhejiang Province, China; University of Maryland, United States of America

## Abstract

The fern *Dicranopteris dichotoma* is an important pioneer species of the understory in Masson pine (*Pinus massoniana*) forests growing on acidic soils in the subtropical and tropical China. To improve our understanding of the role of *D. dichotoma* in nitrogen (N) uptake of these forests, a short-term ^15^N experiment was conducted at mountain ridge (MR, with low N level) and mountain foot (MF, with high N level). We injected ^15^N tracers as ^15^NH_4_, ^15^NO_3_ or^ 15^N-glycine into the soil surrounding each plant at both MR and MF sites. Three hours after tracer injection, the fern *D. dichotoma* took up ^15^NH_4_^+^ significantly faster at MF than at MR, but it showed significantly slower uptake of ^15^NO_3_^−^ at MF than at MR. Consequently, ^15^NO_3_^−^ made greater contribution to the total N uptake (50% to the total N uptake) at MR than at MF, but ^15^N-glycine only contributed around 11% at both sites. Twenty-four hours after tracer injection, *D. dichotoma* preferred ^15^NH_4_^+^ (63%) at MR, whereas it preferred ^15^NO_3_^−^ (47%) at MF. We concluded that the *D. dichotoma* responds distinctly in its uptake pattern for three available N species over temporal and spatial scales, but mainly relies on inorganic N species in the subtropical forest. This suggests that the fern employs different strategies to acquire available N which depends on N levels and time.

## Introduction

Nitrogen (N) is a major limiting element in many terrestrial ecosystems [15], [32]. In the past three decades a large number of plants have been identified to have the capacity to directly take up organic N, mainly in the form of amino acids from soil solution [3], [11], [21], [22]. Therefore, uptake pattern of different N species may be an important mechanism responsible for species coexistence in plant communities[19]. Numerous studies on plant N acquisition of organic and inorganic N species have been conducted in subtropical and tropical forests, most focusing on tree species [1], [27], [28], [29], [36], [37], [38], bryophytes and lichens [6], [34] as well as some epiphytes [8], [35]. While there was one study investigating N acquisition by a tropical fern [39], little is done to study organic and inorganic N uptake by subtropical ferns.

**Figure 1:**
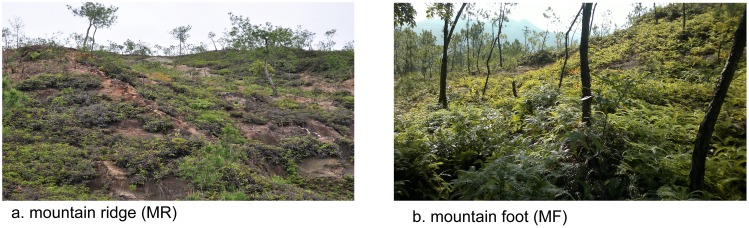
The two sites located along the mountain slope: (a) mountain ridge (MR), (b) mountain foot (MF).

*Dicranopteris dichotoma* is an important terrestrial fern and is widely distributed in southern China as an early-stage colonizer of acidic and oligotrophic soils [9], [40]. *D. dichotoma* is characterized by rapid clonal growth and often forms a dense understory layer in humid subtropical and tropical forests (Figure 1). Numerous studies have suggested that *Dicranopteris* species can influence many ecological processes in these forests, such as soil erosion, nutrient cycling, tree regeneration, and plant community succession [5], [25], [26], [41]. Recently, understory removal experiments showed that the *Dicranopteris*-dominated understory can form favorable soil microclimates and acts as a major driver of soil biota and ecological processes in forest ecosystems [18], [40]. However, N acquisition mechanisms of *D. dichotoma* remain unknown in these tropical forests, and clarification of *D. dichotoma* N uptake patterns could improve the mechanistic understanding of its role in these subtropical and tropical forests.

To investigate organic and inorganic N acquisition by *D. dichotoma*, we selected two different habitats in a subtropical forest (Figure 1): one located at the mountain ridge (MR) and the other located at the mountain foot (MF). Compared to the MF site, the MR site is characterized by heavy soil erosion and relatively lower N availability. Numerous studies have demonstrated that plants under high available N levels show higher N uptake rates [7], [30]. Therefore, we hypothesized that *D. dichotoma* would have greater N uptake capacity at high N level than at low N level. Additionally, we hypothesized that *D. dichotoma* could acquire more nitrate than ammonium and organic N because it is more mobile in soil solution [31]. To test these hypotheses, we conducted a short-term ^15^N labeling experiment in both *D. dichotoma* communities with different available N levels.

## Materials and Methods

### Study Site

This study was carried out at Xingguo Soil Erosion Observation Station (which belongs to the Institute of Geographic Sciences and Natural Resources, Chinese Academy of Sciences) in Xingguo County (26°30′N, 115° 28′E, 80 m above sea level) of Jiangxi Province, southern China, where no additional special permission for the research site was needed given that the site is owned by the institute and a long-term research permission from the local government exists since 1993. Moreover, our study did not involve any endangered or protected species. Two sites were selected along a mountain slope (Figure 1): one was located at the mountain ridge (MR) and the other was located at the foot of the mountain (MF). The understory was dominated by *D. dichotoma* at both sites. The *Pinus massoniana* trees at both sites had been planted more than 20 years before the study. In the MR sites, the trees are very small because of heavily eroded soil. In contrast, the trees grow better in the MF site. Soil was classified as loamy Lixisol and some properties are presented in Table 1, showing higher nutrient concentrations at the MF site than at the MR site.

**Table 1:** Characteristics of topsoil (0–10 cm) of the mountain ridge (MR) and the mountain foot (MF) sites where *Dicranopteris dichotoma* grows.

	Mountain ridge	Mountain foot
Total N (%)	0.037±0.003	0.075±0.019
Organic carbon (%)	0.65±0.10	1.54±0.45
C/N	16.85±1.57	19.47±1.33
pH (H_2_O)	4.54±0.04	4.45±0.04
Soil moisture (%)	8.93±0.50*	18.30±1.00*
Ammonium (µg N g^−1^ dw soil)	5.07±0.61*	14.52±0.79*
Nitrate (µg N g^−1^ dw soil)	0.09±0.03	0.05±0.01
Glycine (µg N g^−1^ dw soil)	0.05±0.01*	0.12±0.03*

Means (±1SE) of six replicates are presented (n = 6). Asterisks indicate significant differences between two sites at *P<*0.05.

Thirty-six similar sized clusters of *D. dichotoma* were randomly selected at both sites. They were divided into three groups with each group including 12 individual plants. Each of the groups was labeled either with ^15^NH_4_^+^ (^15^NH_4_NO_3_, 98.4 atom% ^15^N), ^15^NO_3_^−^ (NH_4_^15^NO_3_, 98.2 atom% ^15^N), and ^15^N-glycine (C_2_H_5_^15^NO_2_, 95.0 atom% ^15^N), by injecting 10 µg N g^−1^ dw soil. The tracers were injected into the soil at 3 cm depth in a pattern representing the three points of a triangle, with 5 cm length between points and the plant at the centre of the triangle. Six clusters were harvested 3 h after the tracer was injected. The remaining 6 clusters for each treatment were harvested 24 h after the tracer was injected. These harvested plants were classified into roots and shoots. An additional 6 clusters were injected with the same amount of water following the same pattern and harvested as the control after 24 h. The roots were put into 0.5 mM CaCl_2_ solution for 30 min. Then, they were rinsed with purified H_2_O and dried at 75°C. These plant materials were weighed for biomass. Dried roots and shoots were ground to a fine powder using a ball mill (MM200, Retsch, Germany) for the measurements of N content and ^15^N/^14^N ratios. Fresh soil (upper 10 cm) was collected for nutrient analysis.

Soil NO_3_^–^N and NH_4_^+^-N were determined in 0.5 M K_2_SO_4_ extracts on an auto-analyzer (AA3, Bran-Luebbe, Germany). Soil glycine concentrations were measured by high performance liquid chromatography (Waters 515, Waters Inc., USA) in the same extracts [23]. Aliquots of ground plant material (about 2 mg) and soil (about 40 mg) were weighed into tin capsules for analysing organic C, total N and ^15^N/^14^N ratios using isotope ratio mass spectrometry (IRMS, MAT 253, Finnigan MAT, Germany), with a Flash EA1112 interfaced by ConFlo III to the IRMS. Soil pH was measured using a glass electrode on a 1∶2 soil-to-water ratio by weight.

### N Uptake Calculation

Atom% excess ^15^N (APE) was calculated as the atom% ^15^N difference between plants from ^15^N treated and from control plants. ^15^N uptake by plants was estimated by calculating the ^15^N excess of each plant part (biomass×%N/100×APE/100 for shoot and root individually) and then summing them up and dividing this number by root biomass and expressed as µg ^15^N g^−1^ dw root h^−1^.

The standard errors of means are presented in figures and tables as a variability parameter. T-test was used to compare the difference in N uptake rates and soil characteristics between sites. Tukey HSD test was used to compare the contribution of three N species to total N uptake between MR and MF. Three-way ANOVA was performed to test the effects of site, N species and time and their interactions on N uptake rates. Data transformation of Ln (data) was applied to meet preconditions of variance homogeneity and normal distribution before ANOVA analysis. All differences were tested for significance at *P<*0.05 and all statistical analysis were performed on SPSS 17.0 software package (SPSS Inc., Chicago, IL, USA).

## Results

**Figure 2:**
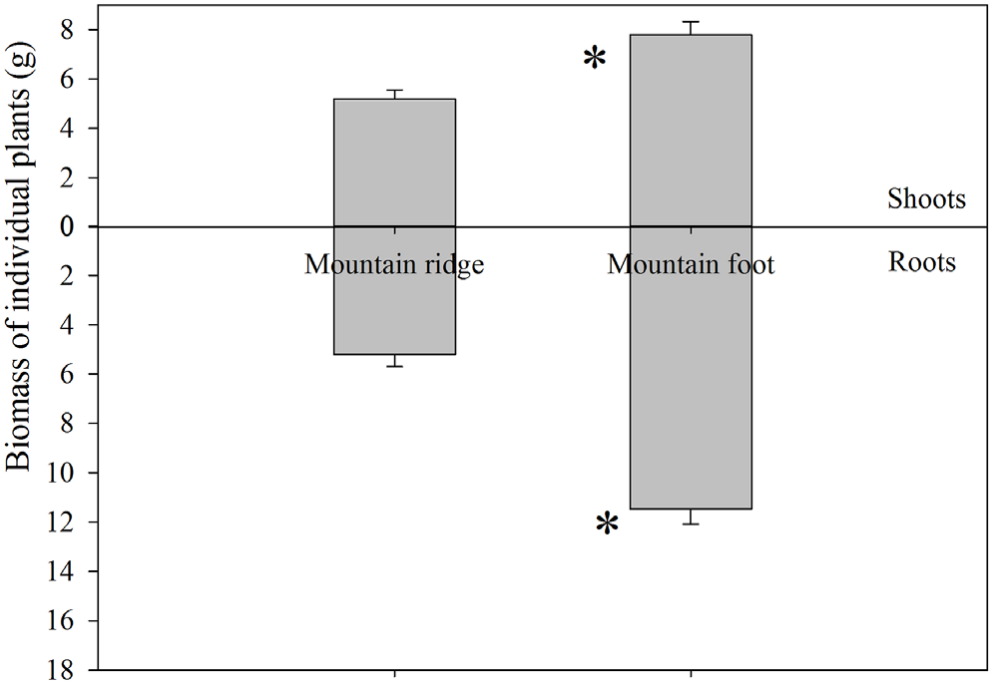
Above-ground and below-ground biomass of the fern *D. dichotoma* located at the mountain ridge (MR) and the mountain foot (MF). Values are means (±1 SE) of 18 replicates. Asterisks indicate significant differences between MR and MF sites at *P<*0.05.

Concentrations of soil organic C and total N were consistently but not significantly higher at MF than MR (Table 1). Compared to MR, concentrations of NH_4_^+^ and glycine were significantly higher at MF (Table 1). NH_4_^+^ was the dominant N species among the three N species, while nitrate and glycine-N concentrations were comparably low at both sites. Total biomass of *D. dichotoma*, including shoots and roots was significantly lower at MR than at MF (*P*<0.05) (Fig. 2). *D. dichotoma* at MR also had significantly lower ratios of root to shoot than at MF (MR vs MF: 1.15±0.11 vs 1.76±0.17) (Fig. 2).

**Figure 3:**
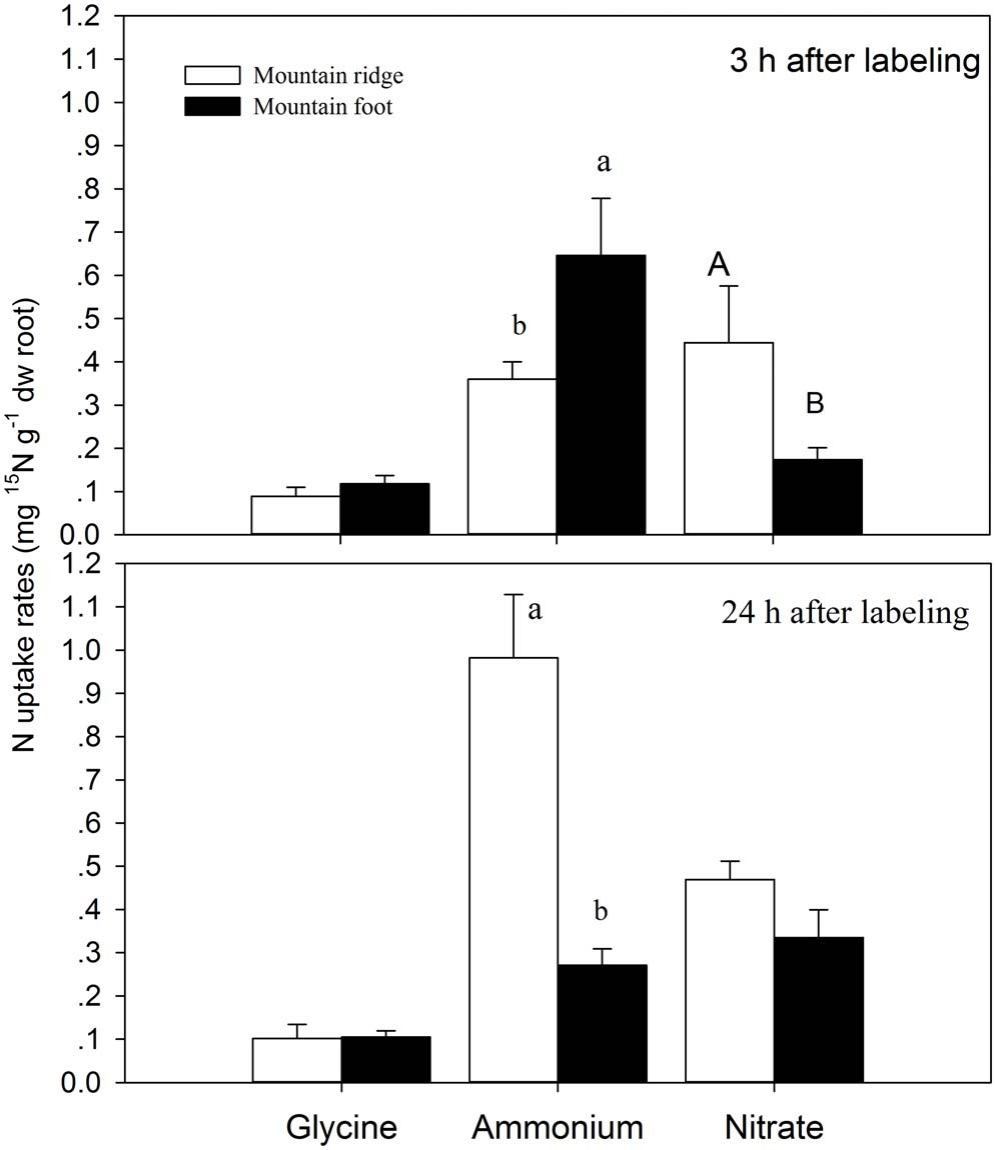
N uptake rates of different N species by the fern *D. dichotoma* at the mountain ridge and mountain foot. Different letters indicate significant differences between the two sites at *P<*0.05.

Results of three-way ANOVA showed significant effects of site, N species and their interactions on N uptake rates but no significant effect of time (Table 2). Three hours after tracer injection, the fern *D. dichotoma* took up ^15^NH_4_^+^ significantly faster (MF vs MR: 0.65±0.13 vs 0.36±0.04 µg ^15^N g^−1^ dw root h^−1^), while it took up less ^15^NO_3_^−^ (MF vs MR: 0.17±0.03 vs 0.44±0.13 µg ^15^N g^−1^ dw root h^−1^) at MF than at MR. Compared to ^15^NH_4_^+^ and ^15^NO_3_^−^, *D. dichotoma* showed considerably lower uptake rates for ^15^N-glycine (MF vs MR: 0.10±0.03 vs 0.09±0.02 µg ^15^N g^−1^ dw root h^−1^) without significant differences between sites (Fig. 3).

**Table 2:** Results of multifactorial ANOVA for the effects of site, N species, time, and their interactions on N uptake rates of *D. dichotoma* in a subtropical forest.

Source of variation	*F*	*P*
Site	5.10	0.03
N species	69.54	0.00
Time	2.16	0.15
Site * N species	4.71	0.01
Site * Time	5.16	0.03
N species * Time	1.12	0.33
Site * N species * Time	7.72	0.00

Twenty-four hours after tracer injection, uptake rates of ^15^N-glycine by *D. dichotoma* did not change at both sites (Fig. 3). Surprisingly, uptake rates of ^15^NH_4_^+^ significantly increased at MR (0.98±0.15 µg ^15^N g^−1^ dw root h^−1^) while they decreased at MF (0.27±0.04 µg ^15^N g^−1^ dw root h^−1^). By comparison, uptake rates of ^15^NO_3_^−^ remained unchanged at MR, but they significantly increased at MF (Fig. 3, T-test, P = 0.034).

**Figure 4:**
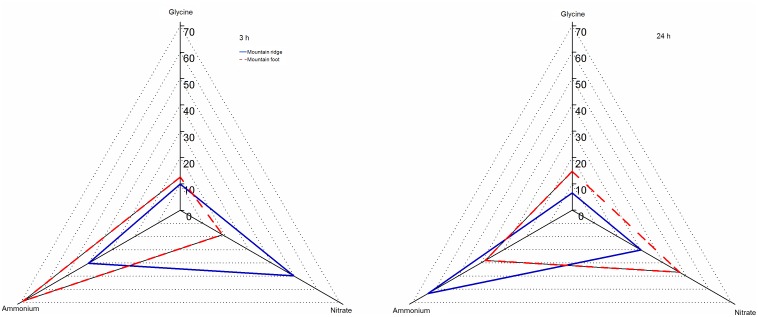
Chemical niche shifts in terms of N acquisition by the fern *D. dichotoma* under two available N levels. The area enclosed by the solid line refers to MR site (low N availability), whereas the dashed line encloses MF site data (high N availability). Values are means (±1SE) of 6 replicates. Asterisks indicate significant differences between mountain ridge and mountain foot MR and MF at *P<*0.05.

Three hours after tracer injection, the fern *D. dichotoma* preferentially took up ^15^NO_3_^−^ at MR (50% of total N uptake) while it preferentially took up ^15^NH_4_^+^ at MF (69% of total N uptake). By comparison, ^15^NH_4_^+^ contributed 40% and 69% to the total N uptake at MR and at MF, respectively. The contribution of ^15^NO_3_^−^ was significantly higher at MR than at MF. The contribution of^ 15^N-glycine was around 11% to the total N uptake at either of the sites (Fig. 4).

Distinct N uptake pattern of *D. dichotoma* was observed at 24 h after tracer injection. *D. dichotoma* preferred ^15^NH_4_^+^ at MR, which contributed 63% to the total N uptake, while the fern preferred ^15^NO_3_^−^ at MF, which contributed about 47% (Fig. 4). The contribution of ^15^N-glycine uptake was significantly higher at MF than at MR (14.8% vs 6.6%) (Fig. 4).

## Discussion

We investigated inorganic and organic N acquisition patterns by the terrestrial fern *D. dichotoma* at two habitats in a subtropical forest using a short-term ^15^N labeling experiment. A previous study in a tropical forest showed that amino acid uptake of a low-light understory terrestrial fern (*Danaea wendlandii*) reached up to about 330 µg N g^−1^ dw root h^−1^ [39]. In this study, the terrestrial fern *D. dichotoma* showed a much lower uptake rate for glycine-N, only around 0.1 µg ^15^N g^−1^ dw root h^−1^. One possible explanation is that Watkins [39] tested uptake of amino acids using excised roots in solution while this study was performed in the field. Roots likely had more opportunity to take up glycine-N in solution than in soil. Available soil N concentrations will be changed through microbial competition and due to microbial mineralization of this organic tracer. Low glycine-N concentrations in the soil (Table 1) also reflect this situation. Besides, the injected soil volume was certainly smaller as the volume of soil extracted to collect the roots afterwards, i.e. many of the collected roots never came into contact with ^15^N. Additionally, excised roots may have been more efficient compared to the roots used in this study, which included more inefficient roots not responsible for nutrient uptake. Nonetheless, it has to be noted that excised roots will loose lose uptake potential over time due to C starvation, because the phloem sugar import has been cut off after root excision.

Our hypothesis that *D. dichotoma* would have greater N uptake capacity at high N level than at low N level was partly supported by our data. Three hours after tracer injection, the fern *D. dichotoma* demonstrated faster uptake rates of ^15^NH_4_^+^ at MF than at MR. One possible explanation could be ascribed to good water status at MF (Table 1), which may enhance the diffusion rate of NH_4_^+^ from soil solution to root surface[2, 10]. However, 24 h after tracer injection, the fern *D. dichotoma* growing at MR showed higher uptake rates for the dominant N form NH_4_^+^. On the basis of the difference in root biomass between MR and MF (Fig. 2), the efficiency of ^15^NH_4_^+^ acquisition by roots at MR is higher than at MF. This indicates that the optimal performance of NH_4_^+^ transporters with high affinity on the root surface [12], [24], [33] could be more important than soil moisture in these soils where NH_4_^+^ is the dominant N species. Besides, strong microbial competition for ^15^NH_4_^+^ could be responsible for low uptake rates by roots at MF [13].

Although soil NO_3_^−^ concentrations were considerably lower compared to NH_4_^+^ (Table 1), the fern *D. dichotoma* showed higher uptake rates for ^15^NO_3_^−^ and its uptake was similar to ^15^NH_4_^+^ uptake rates in some cases (e.g., 3 h after tracer injection at MR and 24 h after tracer injection at MF). One possible explanation is that NO_3_^−^ is more mobile in soil solution [31]. These results indicate that our second hypothesis was partly supported by our observations. This could be explained by the fact that NH_4_^+^ is the dominant N species in both habitats (Table 1), and therefore the fern *D. dichotoma* prefers NH_4_^+^. The preference for NH_4_^+^ was also observed in the tropical terrestrial fern (*D. wendlandii*) despite high NO_3_^−^ concentration [39].

The contribution of the three N species to the total N uptake strongly relies on time and site (Table 2, Fig. 4). Under low N levels (MR), *D. dichotoma* preferentially take up NO_3_^−^ 3 h after tracer injection, but shifted to the dominant N species (NH_4_^+^) 24 h after tracer injection. Under high N levels (MF), *D. dichotoma* preferred the dominant N species (NH_4_^+^) at 3 h after tracer injection and shifted to NO_3_^−^ at 24 h after tracer injection. The distinct uptake pattern for these available N species observed over temporal and spatial scales suggests that the *D. dichotoma* employs different strategies to acquire available N, depending on N levels and time. This could be ascribed to rapid regulation of N uptake modulated by a variety of biotic and abiotic factors in addition to tracer (^15^N) dilution of the available soil N pool, such as the carriers of nitrate, ammonium and amino acids located at the surface of the roots [16], [17], [20]; soil supply rates of available N [4], [14]; delivery of N to the rhizosphere through mass flow and diffusion [10]; as well as competition with soil microorganisms[13]. Further investigations should be focused on how interactions between these biotic and abiotic factors affect N uptake by *D. dichotoma* for a better understanding of the underlying mechanisms.

## Supporting Information

Table S1 **T-test for individual biomass allocation of** ***Dicranopteris dichotoma*** **growing at Mountain Ridge (MR) and Mountain Foot(MF) sites.** Supplementary data, including the characteristics of top soil (0–10 cm), individual biomass allocation of *Dicranopteris dichotoma*, uptake rate of different N species and their contribution to total N uptake rate. And statistical analysis results are also included inside.
(XLS)
